# Trends, Issues and Future Directions of Urban Health Impact Assessment Research: A Systematic Review and Bibliometric Analysis

**DOI:** 10.3390/ijerph19105957

**Published:** 2022-05-13

**Authors:** Wenbing Luo, Zhongping Deng, Shihu Zhong, Mingjun Deng

**Affiliations:** 1 School of Business, Hunan University of Science and Technology, Xiangtan 411201, China; wbluo@hnust.edu.cn (W.L.); 21021502041@mail.hnust.edu.cn (Z.D.); 2 School of Accounting, Hunan University of Technology and Business, Changsha 410205, China; 3 Shanghai National Accounting Institute, Shanghai 201702, China; 4 Big Data and Intelligent Decision Research Center, Hunan University of Science and Technology, Xiangtan 411201, China; mjdeng@hnust.edu.cn

**Keywords:** urban, health impact assessment, bibliometric analysis, CiteSpace, knowledge mapping

## Abstract

Health impact assessment (HIA) has been regarded as an important means and tool for urban planning to promote public health and further promote the integration of health concept. This paper aimed to help scientifically to understand the current situation of urban HIA research, analyze its discipline co-occurrence, publication characteristics, partnership, influence, keyword co-occurrence, co-citation, and structural variation. Based on the ISI Web database, this paper used a bibliometric method to analyze 2215 articles related to urban HIA published from 2012 to 2021. We found that the main research directions in the field were Environmental Sciences and Public Environmental Occupational Health; China contributed most articles, the Tehran University of Medical Sciences was the most influential institution, Science of the Total Environment was the most influential journal, Yousefi M was the most influential author. The main hotspots include health risk assessment, source appointment, contamination, exposure, particulate matter, heavy metals and urban soils in 2012–2021; road dust, source apposition, polycyclic aromatic hydrocarbons, air pollution, urban topsoil and the north China plain were always hot research topics in 2012–2021, drinking water and water quality became research topics of great concern in 2017–2021. There were 25 articles with strong transformation potential during 2020–2021, but most papers carried out research on the health risk assessment of toxic elements in soil and dust. Finally, we also discussed the limitations of this paper and the direction of bibliometric analysis of urban HIA in the future.

## 1. Introduction

The European Union Environmental Impact Assessment (EIA) Directive (2011/92/EU as amended by 2014/52/EU) requires EIAs to consider the effects that a project might have on human health [1]. Over the past two decades, health impact assessment (HIA) practice has expanded across the world [2], HIA has been regarded as an important means and tool for urban planning to promote public health and further promote the integration of health concept. More and more professionals in the fields of spatial planning, transportation planning and public health all over the world are beginning to pay attention to and use HIA tools [3,4,5,6,7]. Many cities around the world set “improving public health” as an important development goal to promote the construction of healthy cities by expanding their understanding and continuously carrying out healthy city planning practice, constructing an HIA theoretical model and carrying out evaluation practice [8,9,10,11,12].

Over the past decade, scholars have increasingly focused on urban HIA research and have issued an increasing number of papers; this makes it difficult for us to grasp the focus of research from thousands of papers, resulting in a major risk of ignoring basic problems and research improvement fields. It will provide an important theoretical basis for researchers, practitioners, decision makers and stakeholders from different backgrounds to carry out urban HIA research and management practice more effectively by presenting the structure, law, and distribution of scientific knowledge in the field of urban HIA. Who have been the famous scholars in urban HIA related research fields? Which countries and institutions have close exchanges in urban HIA related research fields? What are the research topics and development trends in urban HIA related research fields? These problems need to be further analyzed. In order to solve this problem, it is necessary to use a bibliometric analysis method, as this method can better find knowledge status and development trends in a given field [13,14]; additionally use of scientometric software (such as VOSviewer, CoPalRed, Bibexcel, Sci2, VantagePoint, CiteSpace and Online Analysis Platform for Bibliometrics) to realize the visual analysis of citations, so as to reveal how the research field has evolved, the obvious knowledge turning points on the critical path and which topics have attracted people’s attention [15]. However, to the best of our knowledge, there is no bibliometric analysis on urban HIA at a global scale. Therefore, we intuitively displayed the knowledge structure and development trends of urban HIA related research fields through bibliometric analysis, in order to guide scholars and practitioners to determine the research interests and emerging themes of urban HIA, so as to enhance their understanding and evaluation of urban HIA.

This paper is organized as follows. After this introduction, Section 2 provides the primary research materials and methods. Section 3 presents the research findings and analyses. Section 4 presents research-related conclusions.

## 2. Materials and Methods

### 2.1. Data Acquisition

**Figure 1:**
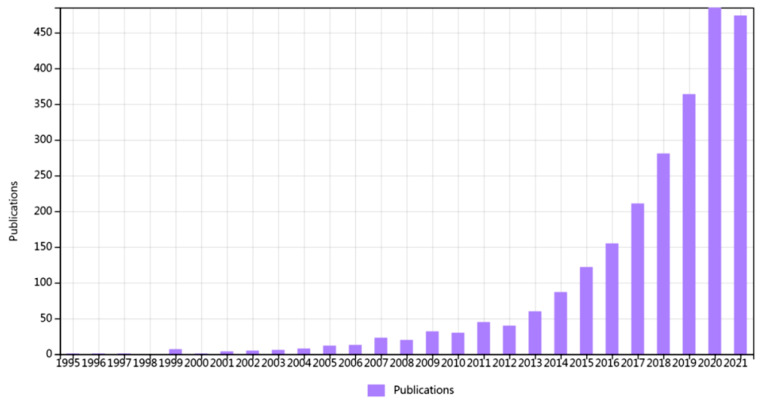
Number of publications over the years.

According to the data resources required by CiteSpace, we took the Web of Science Core Collection as the data collection platform. As people are paying increasing attention to the impact of contaminated sites on the environment and public health, health risk assessment methods were used to describe and quantify the health impact on neighbouring people and guide public health interventions [16]. At the same time, we found that if only “health impact assessment” was used as the search term, the total number of literature studies was less than 400 under the same search conditions. Therefore, this paper juxtaposes “health risk assessment” as a search term. Figure 1 shows the number of papers published each year from 1992 to 2021 according to the retrieval strategy used in this paper. We found that the number of papers published in the early stages was small. CiteSpace software (Version 5.8.R3, Chaomei Chen, Drexel University, Philadelphia, PA, USA) developers pointed out that the longer the literature time span, the poorer the knowledge map. Therefore, this paper focuses on the bibliometric analysis of urban health impact assessment research over the last ten years.

Finally, the search strategy of bibliometric analysis was as follows: This study regards the Web of Science Core Collection as a data-collection platform according to data resources required in CiteSpace; The bibliometric search strategy can be described as the following: TS = (urban OR city OR cities) AND TS = (“health risk assessment” OR “health impact assessment”), Publication years = (1 January 2012 to 14 November 2021), Indexes = (SCI-EXPANDED, SSCI, ESCI), Document types = “Articles”. A total of 2215 publications were selected on 14 November 2021.

### 2.2. Bibliometric Analysis Methods

Bibliometrics is an interdisciplinary study that utilizes mathematics, statistics, and bibliography to quantitatively analyze academic literature [17]. The common methods of bibliometrics include statistical analysis, citation analysis, sharing analysis, etc. In addition, mapping knowledge can show the relationship between the development process and structure of scientific knowledge, focus on the evolution process of a certain knowledge field, and help scholars understand the hot spots, frontiers, and trends of research in this field [18].

Generally, the knowledge of the development status on a research subject is carried out using reviews as systematic or integrative approaches. However, the statistical analysis of journals, authors, countries, institutions and influence can help us quickly grasp the basic information and development status of literature in a certain field [19]. We used the Online Analysis Platform for Bibliometrics (https://bibliometric.com/ (accessed on 16 November 2021)) to conduct publication year, journal, countries and influence analysis, so as to quickly obtain the information of influential institutions, authoritative publications and experts in relevant fields.

Partnership analysis mainly analyzes the relationship between countries, the relationship between institutions and the relationship between authors. We used Online Analysis Platform for Bibliometrics and VOSviewer to analyze the cooperation network among countries, institutions, and authors.

Keyword co-occurrence analysis is an effective method, which can find hot topics and develop research frontiers in specific research fields. In this paper, we made a keyword co-occurrence network map by VOSviewer and monitored hot topics in urban HIA research through keyword co-occurrence network analysis.

Co-citation analysis can map the knowledge structure of a research field, detect the trends in the research field (by the authors engaged in these topics and their interrelationships) and highlight the findings with significant impact [20]. Understanding trends and emerging topics in a research area is important for future and current researchers, policymakers, funding agencies and other stakeholders [21]. CiteSpace enables us to understand a certain field quickly and systematically. CiteSpace can label co-citation clusters and use time-sliced snapshots to form timeliness and pivotal points [14]. In this study, we used CiteSpace (Version 5.8.R3) to find major research areas in the knowledge domain and journal overlay maps. In order to better understand the development of urban HIA research in different periods, we carried out co-citation analysis in two periods: (1) Set a time span from 2012 to 2016, set “years per slice = 1”, “node types = reference”, “Selection Criteria: Select top = 100” and “Pruning = Pathfinder and Pruning sliced networks” in CiteSpace to analyze their intellectual structure and the dynamics of co-citation clusters; (2) Set a time span from 2017 to 2021, set “years per slice = 1”, “node types = reference”, “Selection Criteria: g-index, k = 50” and “Pruning = Pathfinder and Pruning sliced networks” in CiteSpace to analyze their intellectual structure and the dynamics of co-citation clusters.

## 3. Results and Analyses

### 3.1. Discipline Co-Occurrence Analysis

**Figure 2:**
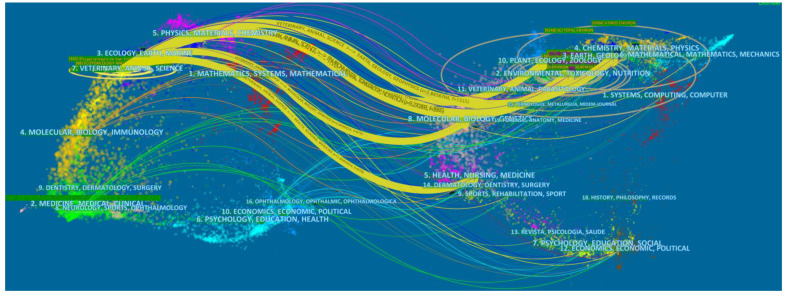
A dual map overlay of literature on urban HIA research.

Via the “dual-map overlay” function of CiteSpace, we can construct the discipline co-occurrence network, as shown in Figure 2. The original map shows more than 10,000 journals indexed in Web of Science, which are divided into different disciplines in different colors and are located in different locations of the source (or left) region and the reference (or right) region. Among them, “source” refers to the relevant papers in a certain field for a certain period of time and “reference” refers to all references of the above papers. For example, the discipline “ECONOMICS, ECONOMIC, POLITICAL” is in lake-blue and it ranks the 10th in the source region and the 12th in the reference region [22]. Then we added a layer containing the 2215 bibliographic records on urban HIA, which became the colorful links between the source region and the reference region.

From Figure 2, the impact of the city and health “7. VETERINARY, ANIMAL, SCIENCE”. As for the distribution of the reference journals, “7. VETERINARY, ANIMAL, SCIENCE” links going to “2. ENVIRONMENTAL, TOXICOLOGY, NUTRITION”, “3. EARTH, GEOLOGY, GEOPHYSICS”, “10. PLANT, ECOLOGY, ZOOLOGY”, “8. MOLECULAR, BIOLOGY, GENETICS” and “5. HEALTH, NURSING, MEDICINE” account for higher percentage. This just reflects that the research on urban HIA is a typical interdisciplinary research field. 

### 3.2. Publication Characteristics Analysis

**Figure 3:**
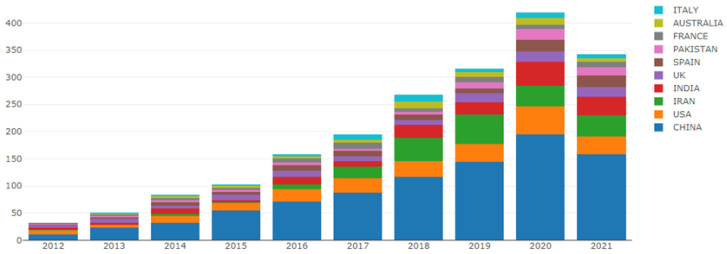
Number of papers published by countries each year.

The top 10 countries in the number of annual publications are shown in Figure 3. Since the Online Analysis Platform for Bibliometrics can only automatically generate the top ten countries, the situation of annual publications in other countries cannot be seen in this figure. The number of articles issued in China, Iran and India has shown a rapid growth trend from 2012 to 2021.

As shown in Table 1: (1) Among the top 10 WOS of categories in the total number of papers published; (2) Among the top 10 publication titles in terms of publication volume; (3) Among the top 10 authors in the number of papers published.

### 3.3. Partnership Analysis

**Figure 4:**
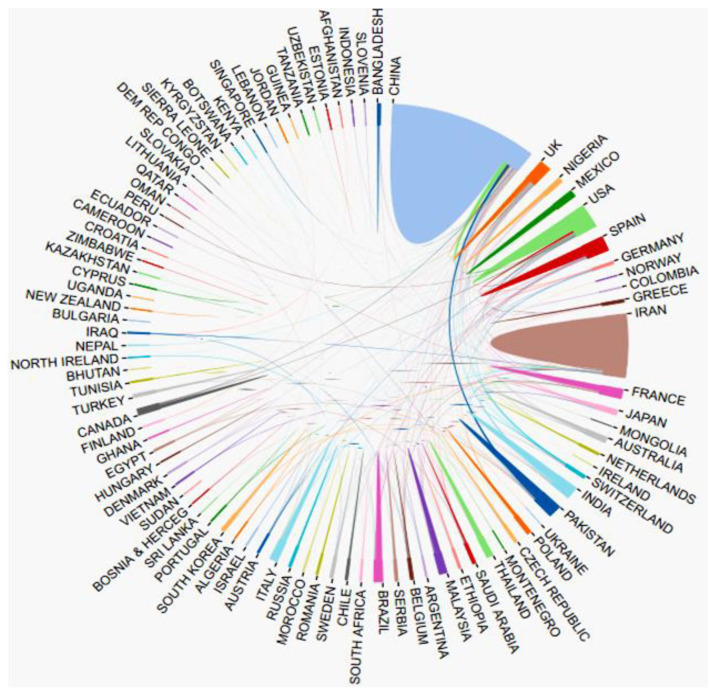
Cooperation between countries.

The cooperative relationship between countries is shown in Figure 4. Among them, authors from CHINA, IRAN, USA, and SPAIN have more cooperation with authors from other countries.

**Figure 5:**
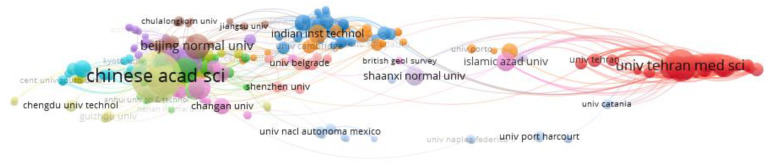
Cooperation between institutions.

The cooperation between institutions is shown in Figure 5. Institutions include universities, research institutes and national research institutions; According to the statistics of the number of articles jointly published by institutions, the organization represented by the red node has a cooperative relationship with the node where the mouse is located. The core of institutional cooperation network mainly includes Chinese Academy of Sciences, Tehran University of Medical Sciences, Beijing Normal University, Indian Institute of Technology, etc.

**Figure 6:**
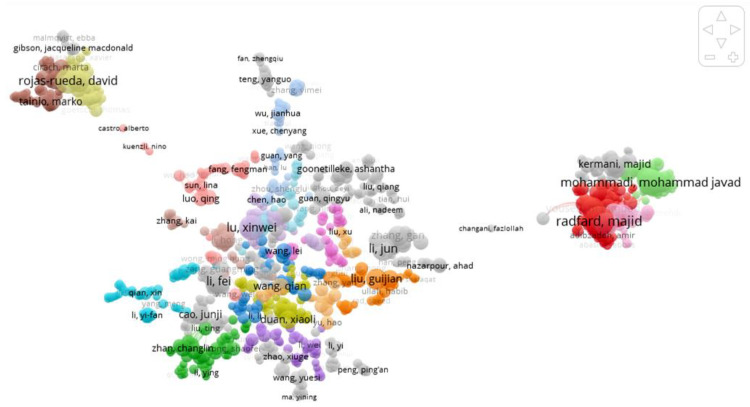
Collaboration between authors.

The cooperation between authors is shown in Figure 6. In the main author cooperation network, the authors who play a key role include Mohammadi M J, Radfard M, Rojas-rueda D, Lu XW, Liu G, Wang Q, Li J, etc.

### 3.4. Influence Analysis

The top 10 institutions with influence are shown in Table 2. Univ Tehran Med Sci, Beijing Normal Univ and Chinese Acad Sci ranked the top three in the total number of references.

The top 10 publication titles with influence are shown in Table 3. “Science of the Total Environment”, “Ecotoxicology and Environmental Safety”, and “Environmental Science and Pollution Research” ranked the top three in the total number of references.

The top 10 authors with influence are shown in Table 4. Yousefi M, Mahvi AH and Lu XW ranked the top three in the total number of references.

### 3.5. Keyword Co-Occurrence Analysis

**Figure 7:**
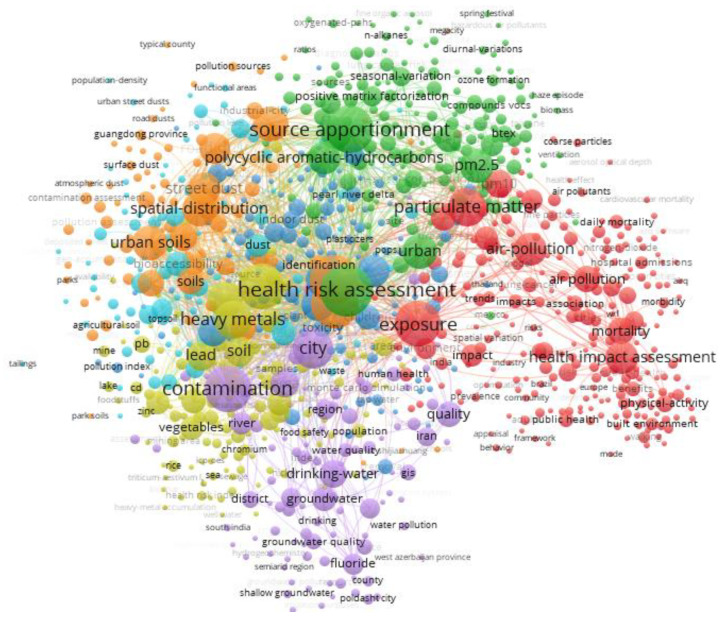
The keyword co-occurrence network.

The knowledge map of keyword co-occurrence can suggest hot topics [5]. We made the keyword co-occurrence network from 2012 to 2021, as shown in Figure 7. Hot keywords include “health risk assessment”, “source appointment”, “contamination”, “exposure”, “particulate matter”, “heavy metals”, “urban soils”, “spatial-distribution”, “pm2.5”, “health impact assessment”, ”polycyclic aromatic-hydrocarbons”, “air-pollution”, etc.

### 3.6. Co-Citation Analysis

The co-citation map of references estimated the scientific relevance of publications [23]. Co-citation cluster analysis was used to explore the research patterns, new trends and their relationships in urban HIA research. Timeline visualization in CiteSpace described clusters through a horizontal timeline. Each cluster was displayed in left-to-right order. The largest cluster was shown at the top of the view. Large nodes or nodes with a red tree structure are particularly interesting because they are either highly referenced, have referenced bursts, or both. Below each timeline, the top three references cited in a particular year are displayed [14].

#### 3.6.1. Timeline View (2012–2016)

**Figure 8:**
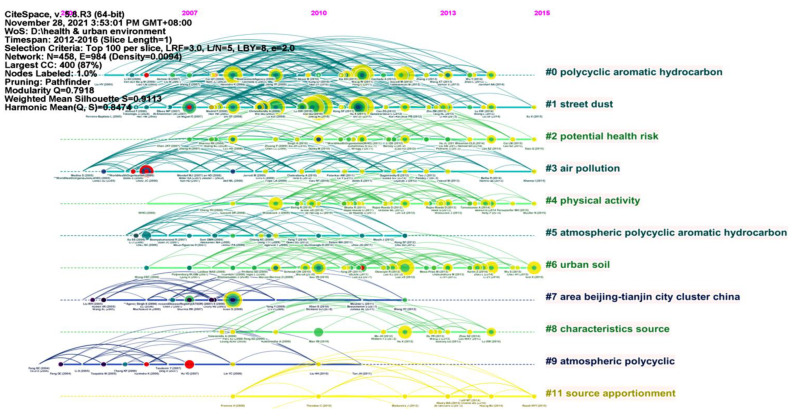
A timeline visualization of clusters on urban HIA research in 2012–2016.

Based on the literature records from 2012 to 2016, we generated a cited reference map with 458 nodes and 984 links (Figure 8). The results show, the mean silhouette (S = 0.9113) and the modularity (Q = 0.7918), that the modular Q value was greater than 0.7, which indicated that it was reasonable for the network to be divided into loosely coupled clusters. 

The five salient clusters were sorted by size (Table 5). The literature with high co-citation frequency became a representative publication in the cluster, which affected the annotation of the cluster and revealed the research frontier. 

Cluster #0 was the largest cluster and represented “polycyclic aromatic hydrocarbons” with 64 members. In the representative publications, Yu et al. (2014) identified the potential sources of polycyclic aromatic hydrocarbons (PAHs) in 87 urban street dust samples from Tianjin as a Chinese megacity and evaluated the risk of PAHs to urban residents using the incremental lifetime cancer risk model [24].

Cluster #1 represented “street dust” with 58 members. In the representative publications, Lu et al. (2014) applied the potential ecological risk index to assess the risk of heavy metals in street dust of cities in China on urban ecosystems and applied the human exposure model to assess the risk of heavy metals to human health. The authors emphasized that further research on street dust exposure parameters and transportation factors was needed to reduce the uncertainty related to risk calculation [25].

Cluster #2 represented “potential health risk” with 45 members. In the representative publications, Fu et al. (2015) estimated the rice’s potential health risk to inhabitants in Fuzhou, China by target hazard quotient (THQ), hazard index (HI) and target cancer risk (TR) [26].

Cluster #3 represented “air pollution” with 42 members. In the representative publications, Lai et al. (2013) systematically evaluated and analyzed the mortality and incidence rate of four typical air pollutants (PM_10_, NO_2_, SO_2,_ and O_3_) in the Chinese population. The authors emphasized that the short-term impact of air pollution could be used for health impact assessment, but the evidence of long-term impact was still insufficient [27].

Cluster #4 represented “physical activity” with 40 members. In the representative publications, Gerike et al. (2016) investigated the determinants of active mobility and the evaluation of measures to increase it through a large-scale longitudinal survey conducted in seven Physical Activity through Sustainable Transport Approaches (PASTA) case study cities. The results would provide data on the health benefits of cycling and/or walking to the WHO’s online health economic assessment tool [28].

**Table 5:** Summary of the largest five clusters in 2012–2016.

Cluster ID	Size	Silhouette	Cluster Label (LLR)	Representative Publication
#0	64	0.894	polycyclic aromatic hydrocarbons	Yu et al. (2014) [24], Jiang et al. (2014) [29], Tuyen et al. (2014) [30], Hoseini et al. (2016) [31], Bulejko et al. (2016) [32], Yue et al. (2015) [33]
#1	58	0.895	street dust	Lu et al. (2014) [25], Han et al. (2016) [34], Sun et al. (2015) [35], Keshavarzi et al. (2015) [36], Han et al. (2016) [37]
#2	45	0.783	potential health risk	Fu et al. (2015) [26], Varol & Davraz (2015) [38], Islam et al. (2015) [39]
#3	42	0.969	air pollution	Lai et al. (2013) [27], Morelli et al. (2016) [40], Arranz et al. (2014) [41], Baccini et al. (2013) [42], Izhar et al. (2016) [43]
#4	40	0.985	physical activity	Gerike et al. (2016) [28], Mansfield & Jacqueline (2015) [44], Gibson et al. (2015) [45], Zapata-Diomedi et al. (2016) [46]

Table 6 lists the detailed information of the top 13 references with strongest citation bursts in 2012–2016. The Sigma metric measures are both citation burstness and structural centrality of a cited reference. Most references were published in top journals. 

#### 3.6.2. Timeline View (2017–2021)

**Figure 9:**
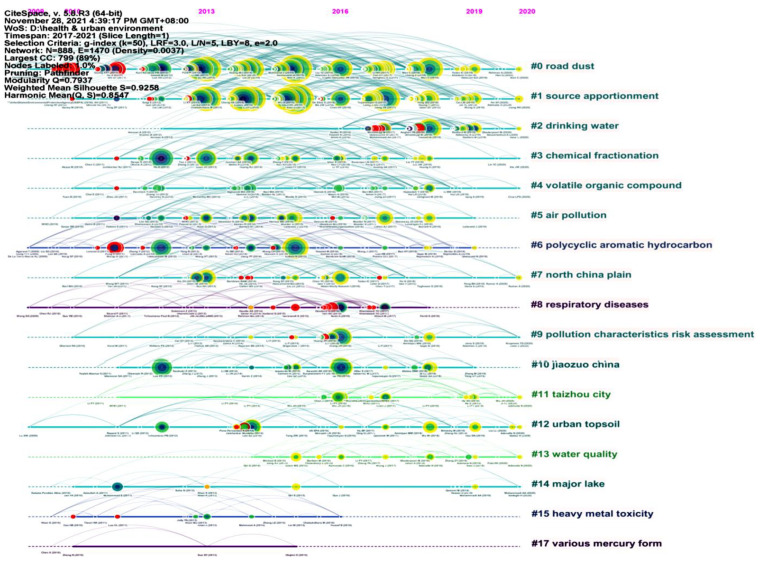
A timeline visualization of clusters on urban HIA research in 2017–2021.

Based on the literature records from 2017 to 2021, we generated a cited reference map with 888 nodes and 1470 links (Figure 9). The results show that with the mean silhouette (S = 0.9258) and the modularity (Q = 0.7937), the modular Q value was greater than 0.7, which indicated that it was reasonable for the network to be divided into loosely coupled clusters. 

The nine salient clusters were sorted by size (Table 7). The literature with high co-citation frequency became a representative publication in the cluster, which affected the annotation of the cluster and revealed the research frontier. 

Cluster #0 represented “road dust” with 121 members. In the representative publications, Moryani et al. (2020) evaluated the pollution level of heavy metals (Cu, Pb, Zn, CD, Ni, Sb, Cr) in five parts of road dust in four different regions of Karachi and Shikarpur by calculating the pollution index enrichment factor and Igeo; the health risk assessment was carried out according to the carcinogenic risk methods and hazard index [47].

Cluster #1 represented “source apportionment” with 119 members. In the representative publications, Cai et al. (2019) studied the levels, distribution, and source apportionment of metals in soils from a typical rapidly developing county, Southern China [48].

Cluster #2 represented “drinking water” with 68 members. In the representative publications, Hamed et al. (2018) assessed the nitrate concentration and also the microbial quality of bottled water in a number of brands produced in the Torbat-e Heydarieh city in 2017 [49].

Cluster #3 represented “chemical fractionation” with 63 members. In the representative publications, Sah et al. (2019) identified the migration potential of metals (As, Cd, Co, Cr, Ni, and Pb) in urban fine particulate matter and assessed the health risks of these metals to infants, young children, children, males, and females. The research conclusions showed that urban aerosols have potential risks to humans [50].

Cluster #4 represented “volatile organic compounds” with 61 members. In the representative publications, Gu et al. (2020) analyzed the chemical characteristics and sources of volatile organic compounds (VOCs) in Tianjin, China, using 1-h resolution VOC-species data between 1 November 2018 and 15 March 2019 [51].

Cluster #5 represented “air pollution” with 52 members. In the representative publications, Lehtomaki et al. (2020) quantified the number of deaths caused by ambient air pollution in Nordic countries using selected assessment tools, identified the main differences and stressed that high spatial resolution should be used to avoid underestimating the health effects of ambient air pollution [52].

Cluster #6 represented “polycyclic aromatic hydrocarbons” with 46 members. In the representative publications, Najmeddin and Keshavarzi (2019) used toxic equivalency factors and increased lifetime cancer risk to assess the health risk of PAHs in PM_10_ and road dust samples [53].

Cluster #7 represented “north China plain” with 45 members. In the representative publications, Zhang et al. (2019) evaluated the potential health risks of PAHs (including gas and particle phases) by combining methods of benzo[a]pyrene equivalent concentration and incremental lifetime cancer risk and identified the potential source regions of PM_2.5_-bound PAHs in Jinan by the Concentration Weighted Trajectory model [54].

Cluster #8 represented “respiratory diseases” with 37 members. In the representative publications, Geravandi et al. (2017) studied the relationship between the number of hospitalized respiratory diseases (including asthma attacks, acute bronchitis, and chronic obstructive pulmonary disease) caused by PM_10_ and normal/dust event days in Ahvaz, Iran, from 2010 to 2012 [55].

**Table 7:** Summary of the largest nine clusters in 2017–2021.

Cluster ID	Size	Silhouette	Cluster Label (LLR)	Representative Publication
#0	121	0.847	road dust	Moryani et al. (2020) [47], Faisal et al. (2021) [56], Chen et al. (2019) [57], Ahamad et al. (2021) [58], Shabanda et al. (2019) [59], Mondal & Singh (2021) [60], Jiang et al. (2018) [61], Othman & Latif (2020) [62], Heidari et al. (2021) [63], Shahab et al. (2020) [64], Wang et al. (2021) [65]
#1	119	0.874	source apportionment	Cai et al. (2019) [48], Duan et al. (2020) [66], Zhang et al. (2021) [67], Li et al. (2021) [68], Sun et al. (2020) [69], Tang et al. (2020) [70]
#2	68	0.982	drinking water	Hamed et al. (2018) [49], Qasemi et al. (2019) [71], Badeenezhad et al. (2021) [72], Radfard et al. (2019) [73], Mirzabeygi et al. (2018) [74]
#3	63	0.962	chemical fractionation	Sah et al. (2019) [50], Long et al. (2021) [75], Jan et al. (2018) [76], Guo et al. (2021) [77], Jiang et al. (2020) [78]
#4	61	0.971	volatile organic compounds	Gu et al. (2020) [51], Li et al. (2020) [79], Ding et al. (2020) [80], Tohid et al. (2019) [81], Xiong et al. (2020) [82], Wang et al. (2020) [83], Li et al. (2020) [84]
#5	52	0.967	air pollution	Lehtomaki et al. (2020) [52], Izquierdo et al. (2020) [85], Luo et al. (2020) [86], Giallouros et al. (2020) [87], Sacks et al. (2018) [88], Sohrabi et al. (2020) [89], Khomenko et al. (2021) [90], Gamarra et al. (2021) [91]
#6	46	0.970	polycyclic aromatic hydrocarbons	Najmeddin et al. (2019) [53], Abbasnejad et al. (2019) [92], Najmeddin et al. (2018) [93], Qishlaqi & Beiramali (2019) [94], Liang et al. (2019) [95]
#7	45	0.904	north China plain	Zhang et al. (2019) [54], Shen et al. (2019) [96], Zhang et al. (2019) [97], Luo et al. (2021) [98], Gao et al. (2019) [99]
#8	37	0.969	respiratory diseases	Geravandi et al. (2017) [55], Khaniabadi et al. (2017) [100]

Table 8 lists the detailed information of the top 30 references with strongest citation bursts in 2017–2021. There are 85 references with the strongest citation bursts in 2017–2021, we chose 30 representative references. The citation burstness and structural centrality of the cited references can be measured by Sigma metric. The early foundational documents and these 85 highly cited documents together constitute the intellectual base of urban HIA research in 2017–2021.

### 3.7. Structural Variation Analysis (SVA)

#### 3.7.1. Articles with Transformative Potentials

The main limitation of citation-based indicators is that they may ignore newly published articles. SVA is a method to focus on the impact of newly published papers on the conceptual structure of related knowledge fields [101]. The SVA program looks for new connections that may change the global structure [102]. The purpose of applying SVA is to evaluate the potential of an article to establish abnormal or unexpected connections between different clusters. From the perspective of scientific discovery theory, many significant contributions come from the idea of crossing borders [14]. To assess the recent papers’ transformative potentials, we used the SVA of CiteSpace—we used 3-year span sliding windows. Table 9 shows a list of articles with a high transformative potential based on the ΔModularity and ΔCluster Linkage. 

#### 3.7.2. Trajectories of Citations across Cluster Boundaries

**Figure 10:**
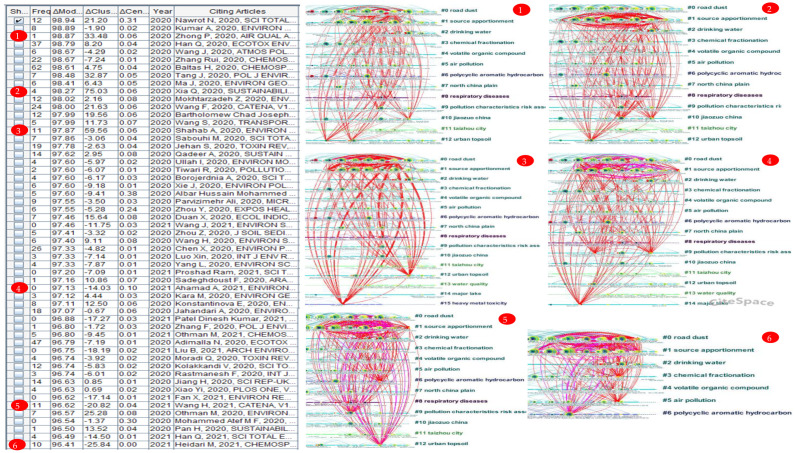
Six examples of articles with high modularity change rates.

There were six examples of articles with high modularity change rates, as shown in Figure 10. The visualization reveals the distribution of the references cited by these articles across different clusters. 

(1) Zhong et al. (2020) analyzed two particle size distributions of heavy metals in street dust from an industrial city, explored their possible sources and assessed their health risks [103]. The papers cited in this paper are mainly distributed in clusters #0, #1, #9, #10, and #12.

(2) Xia et al. (2020) explored the source of six potentially toxic elements (PTEs) in topsoil of three different land use types (residential land, industrial land, and farmland) in Tonghua City and evaluated the ecological risk and human health risk of PTEs in different types of soil [104]. The papers cited in this paper are mainly distributed in clusters #0, #1, #10, and #12.

(3) Shahab et al. (2020) evaluated the pollution level and health risk from heavy metals in road dust collected in three functional areas in the tourist city Guilin using the geoaccumulation index (Igeo), ecological risk index, spatial interpolation, and array-based risk assessment model [64]. The papers cited in this paper are mainly distributed in clusters #0, #1, #12 and #15.

(4) Ahamad et al. (2021) identified the sources of potentially toxic elements (As, Cd, Cr, Cu, Fe, Hg, Mn, Ni, Pb, Zn) of 48 samples of soil and road dust from industrial clusters in the Sonbhadra region of Uttar Pradesh (India) and assessed their human health risks [58]. The papers cited in this paper are mainly distributed in clusters #0, #1, #9 and #14.

(5) Wang et al. (2021) evaluated the pollution characteristics and spatial distribution characteristics of 12 PTEs (Mn, Ni, Cu, Zn, Hg, Cd, As, Cr, Pb, Tl, Co, and Sb) in a large copper smelter in Central China and evaluated the potential ecological and health risks of PTEs by combining with positive matrix decomposition [105]. The papers cited in this paper are mainly distributed in clusters #0, #1, #2, #6, #9, and #12.

(6) Heidari et al. (2021) assessed the related specific source-ecological and health risks of heavy metals (As, Cd, Co, Cr, Cu, Mn, Ni, Pb, and Zn) in road dust in Bandar Abbas (Iran) and its western suburb [63]. The papers cited in this paper are mainly distributed in clusters #0, #1, #3, #5, and #6.

## 4. Discussions

The limitations of this study are shown as follows: 

First, the data only comes from English papers in the WoS Core Collection, resulting in the exclusion of a large number of English documents in Scopus, PubMed/Medline and other databases, as well as a large number of documents in other language countries [106]. It is necessary to conduct bibliometric analysis of urban HIA related literature in other databases and urban HIA related literature in other languages [107], so as to grasp the global research progress more comprehensively on urban HIA. 

Second, as urban HIA is a global topic, the situation of each country is different, so the research content may also be different. In the future, we can select country samples for comparative research, so as to understand the research progress of various countries in the field of urban HIA.

Finally, due to the limitation of the software itself, in the co-citation analysis, some important research topics in the field of urban HIA might have been omitted, for example, the urban HIA practice issues [1,2,108,109,110] and HIA in urban planning [111,112,113]. These important research topics should be employed in a bibliometric analysis in the future.

## 5. Conclusions

This paper used the bibliometric method to analyze the trends, issues and future directions of urban HIA research. We advance the following main conclusions:

First, the main research directions in the field were Environmental Sciences and Public Environmental Occupational Health. 

Second, China contributed most articles; the Univ Tehran Med Sci was the most influential institution; Science of the Total Environment was the most influential journal; Yousefi M was the most influential author.

Third, the main hotspots included health risk assessment, source appointment, contamination, exposure, particulate matter, heavy metals and urban soils in 2012–2021. 

Fourth, the road dust, source apposition, PAHs, air pollution, urban topsoil and north China plain were always hot research topics in 2012–2021. Drinking water and water quality became research topics of great concern in 2017–2021.

Finally, there were 25 articles with strong transformation potential during 2020–2021, but most papers carried out research on health risk assessment of toxic elements in soil and dust.

## Figures and Tables

**Table 1:** Top 10 WOS of categories, publication titles and authors.

WOS Categories	Record Count	Publication Titles	Record Count	Authors	Record Count
Environmental Sciences	1640	Environmental Science and Pollution Research	161	Li J	27
Public Environmental Occupational Health	408	Science of the Total Environment	146	Wang Q	27
Engineering Environmental	233	International Journal of Environmental Research and Public Health	106	Radfard M	25
Water Resources	217	Environmental Geochemistry and Health	101	Rojas-rueda D	24
Toxicology	178	Human and Ecological Risk Assessment	93	Latif MT	21
Meteorology Atmospheric Sciences	115	Ecotoxicology and Environmental Safety	88	Mohammadi MJ	21
Biodiversity Conservation	99	Environmental Pollution	81	Lu XW	20
Multidisciplinary Sciences	76	Chemosphere	80	Zhang H	19
Geosciences Multidisciplinary	58	Atmospheric Environment	43	Zhang JQ	18
Chemistry Analytical	49	Environmental Research	43	Li F	17

**Table 2:** Top 10 institutions with influence.

Institution Name	Total Number of Articles	Total References	Average Cited Times	Total Number of First Authors	Number of Citations of the First Author	Average Citation of the First Author
Univ Tehran Med Sci	107	801	7.49	30	276	9.20
Beijing Normal Univ	56	617	11.02	35	301	8.60
Chinese Acad Sci	215	539	2.51	84	217	2.58
Ahvaz Jundishapur Univ Med Sci	106	437	4.12	20	109	5.45
Univ Kebangsaan Malaysia	64	356	5.56	19	112	5.89
Chinese Res Inst Environm Sci	53	260	4.91	19	171	9.00
Shiraz Univ	25	256	10.24	15	154	10.27
China Univ Geosci	54	211	3.91	26	90	3.46
Zhejiang Univ	30	200	6.67	12	157	13.08
Shahid Beheshti Univ Med Sci	50	184	3.68	3	6	2.00

**Table 3:** Top 10 publication titles with influence.

Publication Titles	Total Number of Articles	Total References	Average Cited Times
Science of the Total Environment	146	742	5.08
Ecotoxicology and Environmental Safety	88	565	6.42
Environmental Science and Pollution Research	147	429	2.92
Chemosphere	80	421	5.26
Human and Ecological Risk Assessment	93	257	2.76
Environmental Geochemistry and Health	90	242	2.69
Atmospheric Environment	43	241	5.60
Environmental Pollution	81	195	2.41
Environment International	41	187	4.56
International Journal of Environmental Research and Public Health	106	179	1.69

**Table 4:** Top 10 authors with influence.

Author	Total Number of Articles	Total References	Average Cited Times	Total Number of First Authors	Number of Citations of the First Author
Yousefi M	15	207	13.8	5	79
Mahvi AH	14	186	13.29	0	0
Lu XW	20	157	7.85	3	31
Keshavarzi B	13	148	11.38	3	71
Nabizadeh R	10	141	14.1	0	0
Radfard M	19	139	7.32	5	37
Teng YG	6	134	22.33	0	0
Rojas-Rueda D	24	127	5.29	3	34
Chen HY	5	127	25.4	2	124
Wang YY	9	125	13.89	1	0

**Table 6:** Top 13 references with strongest citation bursts in 2012–2016. The black line represents the year of citation burstness of the paper.

Title	Strength	Begin	End	2012–2016
Use of health impact assessment in the United States: 27 case studies, 1999–2007 (Dannenberg, 2008)	3.87	2012	2013	▃▃▂▂▂
Health effects of fine particulate air pollution: Lines that connect (Chow et al., 2006)	3.22	2012	2014	▃▃▃▂▂
Seasonal and site-specific variation in vapour and aerosol phase PAHs over Flanders (Belgium) and their relation with anthropogenic activities (Ravindra et al., 2006)	2.25	2012	2014	▃▃▃▂▂
Use of health impact assessment in incorporating health considerations in decision making (Davenport et al., 2006)	2.21	2012	2013	▃▃▂▂▂
The 2005 world health organization reevaluation of human and mammalian toxic equivalency factors for dioxins and dioxin-like compounds (den Berg et al., 2006)	2.21	2012	2013	▃▃▂▂▂
Geochemistry and risk assessment of street dust in Luanda, Angola: A tropical urban environment (Ferreira-Baptista & Migu et al., 2005)	2.21	2012	2013	▃▃▂▂▂
Risk-based evaluation of the exposure of children to trace elements in playgrounds in Madrid (Spain) (De Miguel et al., 2007)	2.84	2013	2016	▂▃▃▃▃
Health risk assessment for traffic policemen exposed to polycyclic aromatic hydrocarbons (PAHs) in Tianjin, China (Hu et al., 2007)	2.33	2013	2014	▂▃▃▂▂
Apheis: Health Impact Assessment of Long-term Exposure to PM_2.5_ in 23 European Cities (Boldo et al., 2006)	1.94	2013	2014	▂▃▃▂▂
The status of soil contamination by semivolatile organic chemicals (SVOCs) in China: A review (Cai et al., 2008)	1.94	2013	2014	▂▃▃▂▂
Emission of polycyclic aromatic hydrocarbons in China (Xu et al., 2006)	1.94	2013	2014	▂▃▃▂▂
Probabilistic risk assessment for personal exposure to carcinogenic polycyclic aromatic hydrocarbons in Taiwanese temples (Liao et al., 2006)	1.94	2013	2014	▂▃▃▂▂
Distribution, availability and sources of trace metals in different particle size fractions of urban soils in Hong Kong: Implications for assessing the risk to human health (Luo et al., 2011)	1.95	2014	2016	▂▂▃▃▃

**Table 8:** Top 30 references with strongest citation bursts in 2017–2021. The black line represents the year of citation burstness of the paper.

Title	Strength	Begin	End	2012–2021
Health risk assessment of heavy metal exposure to street dust in the zinc smelting district, Northeast of China (Zheng et al., 2010)	16.11	2017	2018	▃▃▂▂▂
A review of heavy metal contaminations in urban soils, urban road dusts and agricultural soils from China (Wei et al., 2010)	12.64	2017	2018	▃▃▂▂▂
Multivariate statistical analysis of heavy metals in street dust of Baoji, NW China (Lu et al., 2010)	9.56	2017	2018	▃▃▂▂▂
Study of ground-level ozone and its health risk assessment in residents in Ahvaz City, Iran during 2013 (Yari et al., 2016)	6.88	2017	2018	▃▃▂▂▂
Health risk assessment of abandoned agricultural soils based on heavy metal contents in Hong Kong, the world’s most populated city (Luo et al., 2011)	6.49	2017	2018	▃▃▂▂▂
A comparative study of health risk of potentially toxic metals in urban and suburban road dust in the most populated city of China (Shi et al., 2011)	5.97	2017	2019	▃▃▃▂▂
Polycyclic aromatic hydrocarbons (PAHs) in urban surface dust of Guangzhou, China: Status, sources and human health risk assessment (Wang et al., 2011)	5.46	2017	2019	▃▃▃▂▂
Bioaccessibility and health risk of arsenic, mercury and other metals in urban street dusts from a mega-city, Nanjing, China (Hu et al., 2011)	5.46	2017	2019	▃▃▃▂▂
Integrating hierarchical bioavailability and population distribution into potential eco-risk assessment of heavy metals in road dust: A case study in Xiandao District, Changsha city, China (Huang et al., 2016)	5.35	2017	2018	▃▃▂▂▂
An evaluation of hospital admission respiratory disease attributed to sulfur dioxide ambient concentration in Ahvaz from 2011 through 2013 (Goudarzi et al., 2016)	4.96	2017	2018	▃▃▂▂▂
Heavy metals exposure of children from stairway and sidewalk dust in the smelting district, northeast of China (Zheng et al., 2010)	4.96	2017	2018	▃▃▂▂▂
Polycyclic aromatic hydrocarbons in urban soils of Beijing: Status, sources, distribution and potential risk (Peng et al., 2011)	4.94	2017	2019	▃▃▃▂▂
Multivariate and geostatistical analyses of the spatial distribution and sources of heavy metals in agricultural soil in Dehui, Northeast China (Sun et al., 2013)	4.58	2017	2018	▃▃▂▂▂
Exposure to PM_10_, NO_2_ and O_3_ and impacts on human health (Khaniabadi et al., 2017)	4.2	2017	2018	▃▃▂▂▂
Cardiovascular and respiratory mortality attributed to ground-level ozone in Ahvaz, Iran (Goudarzi et al., 2015)	4.2	2017	2018	▃▃▂▂▂
Impact of Middle Eastern Dust storms on human health (Khaniabadi et al., 2017)	4.2	2017	2018	▃▃▂▂▂
Heavy metal contamination and health risk assessment in drinking water of Sistan and Baluchistan, Southeastern Iran (Mirzabeygi et al., 2017)	5.18	2018	2019	▂▃▃▂▂
The concentration data of fluoride and health risk assessment in drinking water in the Ardakan city of Yazd province, Iran (Mirzabeygi et al., 2018)	4.75	2018	2019	▂▃▃▂▂
Drinking water quality and human health risk in Charsadda district, Pakistan (Khan et al., 2013)	3.16	2018	2019	▂▃▃▂▂
Risk assessment and implication of human exposure to road dust heavy metals in Jeddah, Saudi Arabia (Shabbaj et al., 2018)	3.16	2018	2019	▂▃▃▂▂
Association of Hypertension, Body Mass Index and Waist Circumference with Fluoride Intake; Water Drinking in Residents of Fluoride Endemic Areas, Iran (Yousefi et al., 2018)	3.16	2018	2019	▂▃▃▂▂
Source apportionment of atmospheric PM_2.5_-bound polycyclic aromatic hydrocarbons by a PMF receptor model. Assessment of potential risk for human health (Callen et al., 2014)	2.87	2018	2019	▂▃▃▂▂
Sources identification of heavy metals in urban topsoil from inside the Xi’an Second Ringroad, NW China using multivariate statistical methods (Chen et al., 2012)	2.87	2018	2019	▂▃▃▂▂
Levels, sources and health risks of carbonyls and BTEX in the ambient air of Beijing, China (Zhango et al., 2012)	3.53	2019	2021	▂▂▃▃▃
Spatial variation and probabilistic risk assessment of exposure to fluoride in drinking water (Fallahzadeh et al., 2018)	3.31	2019	2021	▂▂▃▃▃
Probabilistic risk assessment of Chinese residents’ exposure to fluoride in improved drinking water in endemic fluorosis areas (Zhang et al., 2017)	3.09	2019	2021	▂▂▃▃▃
Investigation of outdoor BTEX: Concentration, variations, sources, spatial distribution and risk assessment (Miri et al., 2016)	3.09	2019	2021	▂▂▃▃▃
Inhalation exposure and related health risks of BTEX in ambient air at different microenvironments of a terai zone in north India (Masih et al., 2016)	2.87	2019	2021	▂▂▃▃▃
Pollution, ecological-health risks and sources of heavy metals in soil of the northeastern Qinghai-Tibet Plateau (Wu et al., 2018)	2.65	2019	2021	▂▂▃▃▃
Trends of BTEX in the central urban area of Iran: A preliminary study of photochemical ozone pollution and health risk assessment (Hajizadeh et al., 2018)	2.2	2019	2021	▂▂▃▃▃

**Table 9:** Some of the articles with the strongest transformative potentials, M is ΔModularity, C-L is for ΔCluster Linkage, C-D is for ΔCentrality Divergence.

Year	M	C-L	C-D	Title
2020	98.94	21.2	0.31	The effects of urban vehicle traffic on heavy metal contamination in road sweeping waste and bottom sediments of retention tanks (Nawrot et al., 2020)
2020	98.87	33.48	0.06	Contamination characteristics of heavy metals in particle size fractions from street dust from an industrial city, Central China (Zhong et al., 2020)
2020	98.27	75.03	0.06	Pollution, sources and human health risk assessment of potentially toxic elements in different land use types under the background of industrial cities (Xia et al., 2020)
2020	97.99	19.56	0.06	Characteristics and health risk assessment of heavy metals in street dust for children in Jinhua, China (Bartholomew et al., 2020)
2020	97.87	59.56	0.06	Pollution characteristics and toxicity of potentially toxic elements in road dust of a tourist city, Guilin, China: Ecological and health risk assessment (Shahab et al., 2020)
2020	97.46	15.64	0.08	Geostatistical mapping and quantitative source apportionment of potentially toxic elements in top- and sub-soils: A case of suburban area in Beijing, China (Duan et al., 2020)
2020	97.4	9.11	0.08	Spatial distribution of pollution characteristics and human health risk assessment of exposure to heavy elements in road dust from different functional areas of Zhengzhou, China (Wang et al., 2020)
2020	97.16	10.86	0.07	Hazard, ecological and human health risk assessment of heavy metals in street dust in Dezful, Iran (Sadeghdoust et al., 2020)
2021	97.13	−14.03	0.10	Potentially toxic elements in soil and road dust around Sonbhadra industrial region, Uttar Pradesh, India: Source apportionment and health risk assessment (Ahamad et al., 2021)
2020	97.11	12.5	0.06	Pollution status and human health risk assessment of potentially toxic elements and polycyclic aromatic hydrocarbons in urban street dust of Tyumen city, Russia (Konstantinova et al., 2020)
2021	96.88	−17.27	0.03	Contamination and health risk assessment of potentially harmful elements associated with roadside dust in Dhanbad India (Patel and Jain, 2021)
2021	96.75	−18.19	0.02	Heavy metals in indoor dust across China: Occurrence, sources and health risk assessment (Liu et al., 2021)
2021	96.62	−20.82	0.04	A comprehensive exploration of risk assessment and source quantification of potentially toxic elements in road dust: A case study from a large Cu smelter in central China (Wang et al., 2021)
2021	96.62	−17.14	0.01	Risk and sources of heavy metals and metalloids in dust from university campuses: A case study of Xi’an, China (Fan et al., 2021)
2020	96.57	25.28	0.08	Pollution characteristics, sources and health risk assessments of urban road dust in Kuala Lumpur City (Othman and Latif, 2020)
2021	96.49	−14.5	0.01	Pollution effect assessment of industrial activities on potentially toxic metal distribution in windowsill dust and surface soil in central China (Han et al., 2021)
2021	96.41	−25.84	0	Heavy metal pollution of road dust in a city and its highly polluted suburb; quantitative source apportionment and source-specific ecological and health risk assessment (Heidari et al., 2021)
2021	96.33	−14.85	0.04	Spatio-temporal distribution and source identification of heavy metals in particle size fractions of road dust from a typical industrial district (Zhu et al., 2021)
2021	95.71	−17.24	0.01	Contamination, distribution and health risk assessment of risk elements in topsoil for amusement parks in Xi’an, China (Guo et al., 2021)
2021	95.58	−9.36	0.04	Urban street dust in the Middle East oldest oil refinery zone: Oxidative potential, source apportionment and health risk assessment of potentially toxic elements (Naraki et al., 2021)
2021	94.97	−13.77	0.06	Water quality and health risk assessment based on hydrochemical characteristics of tap and large-size bottled water from the main cities and towns in Guanzhong Basin, China (Deng et al., 2021)
2021	94.77	−28.26	0.01	Human health risk assessment of heavy metals in the urban road dust of Zhengzhou metropolis, China (Faisal et al., 2021)
2021	94.75	−14.34	0.08	Pollution evaluation, human health effect and tracing source of trace elements on road dust of Dhanbad, a highly polluted industrial coal belt of India (Mondal and Singh, 2021)
2021	92.61	−30.86	0.08	Status, spatial distribution and health risk assessment of potentially harmful element from road dust in steel industry city, China (Wang et al., 2021)
2021	91.84	−17.22	0.01	Pollution, human health risk assessment and spatial distribution of toxic metals in urban soil of Yazd City, Iran (Soltani-Gerdefaramarzi et al., 2021)

## Data Availability

Not applicable.
